# Formulation Development, Process Optimization, and *In Vitro* Characterization of Spray-Dried Lansoprazole Enteric Microparticles

**DOI:** 10.3797/scipharm.1501-08

**Published:** 2015-07-29

**Authors:** Chintan Vora, Riddhish Patadia, Karan Mittal, Rajashree Mashru

**Affiliations:** Pharmacy Department, Faculty of Technology and Engineering, The M.S. University of Baroda, Kalabhavan, Vadodara-390001, Gujarat State, India

**Keywords:** Microparticles, Spray drying, Process optimization, Gastric resistance, Particle size

## Abstract

This research focuses on the development of enteric microparticles of lansoprazole in a single step by employing the spray drying technique and studies the effects of variegated formulation/process variables on entrapment efficiency and *in vitro* gastric resistance. Preliminary trials were undertaken to optimize the type of Eudragit and its various levels. Further trials included the incorporation of plasticizer triethyl citrate and combinations of other polymers with Eudragit S 100. Finally, various process parameters were varied to investigate their effects on microparticle properties. The results revealed Eudragit S 100 as the paramount polymer giving the highest gastric resistance in comparison to Eudragit L 100-55 and L 100 due to its higher pH threshold and its polymeric backbone. Incorporation of plasticizer not only influenced entrapment efficiency, but diminished gastric resistance severely. On the contrary, polymeric combinations reduced entrapment efficiency for both sodium alginate and glyceryl behenate, but significantly influenced gastric resistance for only sodium alginate and not for glyceryl behenate. The optimized process parameters were comprised of an inlet temperature of 150°C, atomizing air pressure of 2 kg/cm^2^, feed solution concentration of 6% w/w, feed solution spray rate of 3 ml/min, and aspirator volume of 90%. The SEM analysis revealed smooth and spherical shape morphologies. The DSC and PXRD study divulged the amorphous nature of the drug. Regarding stability, the product was found to be stable under 3 months of accelerated and long-term stability conditions as per ICH Q1A(R2) guidelines. Thus, the technique offers a simple means to generate polymeric enteric microparticles that are ready to formulate and can be directly filled into hard gelatin capsules.

## Introduction

Lansoprazole (LSP), a BCS Class II compound, belongs to the category of compounds called proton pump inhibitors (PPIs). They have been explicitly employed in the treatment of gastroesophageal reflux disease, erosive esophagitis, heartburn, Zollinger-Ellison syndrome, and the long-term treatment of pathological hypersecretory conditions [1–3]. Additionally, LSP has an inhibitory effect against *Helicobacter pylori* which contributes in augmenting the effect of antibiotics in the treatment of infection [4, 5]. Regarding their mechanism, PPIs get converted into the active sulfenamide metabolite by the acidic environment of parietal cells which further reacts with cysteins of the enzyme H+/K+ ATPase. This causes inactivation of the sulphydryl group of the proton pump, thereby reducing the hydrogen ion concentration. This conversion of LSP into the active form should occur inside the gastric cells, consequently it should be absorbed in an intact form from the intestinal tract [1, 3, 6].

It is well-recognized that the LSP and other PPIs are susceptible to degradation in acidic media. The rate of degradation decreases with a simultaneous increase in pH [7–9]. About its pharmacokinetics, the absolute bioavailability is > 80%, its plasma half-life is 1.5 hours, its time to peak plasma level is 1.7 hours, and its protein binding is about 97% [1, 3]. However, wide intersubject variation has been observed in its bioavailability which may be attributed to genotype variation of CYP2C19, possible degradation by the gastric acid, and limited solubility in water [10].

Therefore, there is a necessity to develop a system which protects LSP from acidic pH and also addresses its solubility issues. Thus, the research envisaged focuses on the preparation of enterosoluble microparticles by the spray drying procedure for its enteric delivery and also improvement of its solubility. The developed microparticles will have all the advantages of multiparticulate systems like uniform distribution in the gastrointestinal tract, have less affected gastric emptying rate and gastric transit time, be less susceptible to dose dumping, and attain more constant plasma levels, etc. [11, 12]. Moreover, it would also have benefits like production via a continuous one-step process and prudent prospects of scale-up. As enteric polymers contain an acidic functional group, it may degrade an acid-labile drug like LSP. The proposed work encompasses priorly dispersing the enteric polymers at a higher pH to make it soluble [13]. Moreover, the drug may remain in an amorphous state in the microparticles which will ultimately help in improving its solubility and dissolution [14–16].

The specific aims of this work are: (1) understanding the influence of the polymer and/or polymer combinations on *in vitro* gastric resistance and entrapment efficiency, (2) to optimize the process parameters to get the desired attributes, (3) to evaluate the physicochemical properties, drug release studies, and stability studies for the prepared microparticles.

## Experimental

### Materials

Lansoprazole was generously gifted by Cadila Healthcare Ltd., Ahmedabad, India. Eudragit L 100-55, L 100, and S 100 (Evonik Ind., Mumbai, India) and glyceryl behenate (Compritol 888 ATO) (Gatteffose, India) were kindly gifted from the indicated sources. Sodium alginate, potassium bromide (KBr), and triethyl citrate (S. D. Fine Chem Pvt. Ltd., India) were purchased from the indicated source. All other ingredients, chemicals, and reagents were of analytical grade and were used as received.

### Preparation of Microparticles by Spray Drying

The spray drying operation was performed using a laboratory-scale spray dryer (LU-227, Labultima, Mumbai, India). Firstly, the polymer was dispersed in half the quantity of required water which was subsequently neutralized by the addition of sodium hydroxide (NaOH) till pH 9 was obtained. pH 9 was selected as it was reported to be the most stable pH for LSP where its degradation is the minimum [7]. To this neutralized solution of enteric polymer, finely ground LSP (sifted through ASTM # 150) was slowly added and dispersed into it. Finally, the water was added to adjust the desired feed solution concentration.

### Evaluation of Microparticles

#### Entrapment Efficiency

Initially, the microparticles were washed with acetonitrile to remove the free drug. Then the microparticles were dried in a vacuum oven (Ohmkar Equipments, India) at 30°C overnight. Subsequently, microparticles equivalent to 30 mg LSP were weighed and transferred to a 100-ml volumetric flask. To this, 60 ml of 0.1 N NaOH was added and sonicated for 30 minutes. Finally, the volume was made up with pH 6.6 buffer. Further dilutions were made with diluent; 45:55:0.1 v/v mixture of water: acetonitrile: triethylamine adjusted to pH 10. A validated RP-HPLC (Shimadzu, Kyoto, Japan) method was used for drug content measurement. Analysis was performed on the Phenomenex C18 column (250 mm x 4.6 i.d., 5 microns) at 1.0 ml/min flow rate with a 45:55:0.1 v/v mixture of water: acetonitrile: triethylamine adjusted to pH 7 at 285 nm detection wavelength. To determine the entrapment efficiency, the following practical relationship was used [17]:


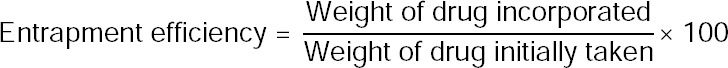


#### In Vitro Gastric Resistance

The gastric resistance study was evaluated using the United States Pharmacopeia (USP) 29 Type II Apparatus (VDA 6-DR, Veego Instruments Corporation, Mumbai, India) using 500 ml of 0.1 N hydrochloric acid (HCl) at 75 rpm rotation speed and 37°C ± 0.5°C temperature for 60 minutes. Here, prior to performing dissolution in 0.1 N HCL, the assay was carried out for the microparticles. After 1 hr, the sample was carefully removed and assayed again. The difference in the assay gives the gastric resistance or the amount of drug that remained protected in acidic media. The higher the amount of drug degraded in acidic media, consequently, will reflect a greater difference in assay value of the microparticles (before and after dissolution). Hence, a good formulation will exhibit a low gastric resistance value and a bad formulation has vice versa. A validated RP-HPLC (Shimadzu, Kyoto, Japan) method was used for drug content measurement as discussed above.

#### In Vitro Drug Release Study

Dissolution was carried out using the USP 29 Type II Apparatus (VDA 6-DR, Veego Instruments Corporation, Mumbai, India) using 500 ml of 0.1 N HCl at 75 rpm rotation speed and 37°C±0.5°C temperature for 60 minutes as mentioned in the USP [18] followed by a buffer stage consisting of 900 ml phosphate buffer pH 7.4 for 60 minutes. Samples were removed from the dissolution media at different time intervals and an equal volume of dissolution medium was replaced at each sampling time to maintain a constant media volume. Samples withdrawn were filtered through a 0.22 μm membrane filter and then analyzed for drug release by the RP-HPLC method as described in the above section.

#### Gastric Resistance and In Vitro Drug Release in Modified Acid Stage Medium pH 4.5

The enteric performance is usually carried out in acidic media at pH 1.2. However, it is reported that stomach pH for PPIs on multiple regimens is >4 and hence, the enteric performance of various enteric polymers must be verified at higher pH or biorelavent media better simulating the gastric environment have been suggested [19]. Thus, microparticles were investigated for gastric resistance in modified acidic media consisting of a pH 4.5 acetate buffer followed by *in vitro* drug release in phosphate buffer pH 7.4 by the same procedure as described for *in vitro* drug release.

#### Particle Size Analysis

The average particle diameter and size distribution of microparticles were determined by using Malvern (Mastersizer 2000, Malvern Instruments, UK) after its dispersion in iso-octane. An aliquot of the microparticle suspension was then added into the small volume recirculation unit and circulated at 3500 rpm. Each sample was measured in triplicate in the analysis.

#### Scanning Electron Microscopy (SEM) Study

The purpose of the SEM study was to obtain topographical images of microparticles. SEM photographs were taken using a scanning electron microscope (JEOL JSM-6380LV, USA) at the required magnification at room temperature after they were gold-sputtered. The acceleration voltage used was 10 kV, with the secondary electron image as the detector.

#### Differential Scanning Calorimetry (DSC) Study

DSC of pure LSP, a physical mixture of the drug and Eudragit S 100, blank microparticles, and drug-loaded microparticles was obtained using an automatic thermal analyzer system (DSC-60, Shimadzu, Japan). The (3–5 mg) sample was crimped in a standard aluminum pan and heated from 40°C to 250°C at a heating rate of 5°C/min under constant purging of dry nitrogen at 40 ml/min. Temperature calibration was performed using indium as the standard. An empty pan, sealed in the same way as the sample, was used as the reference.

#### Fourier Transform Infrared (FTIR) Spectroscopy Study

The FTIR (Bruker, USA) spectra of the pure drug, physical mixture of the drug and Eudragit S 100, blank microparticles, and drug-loaded microparticles were investigated using the KBr disk method. In brief, the procedure involved mixing a total of 2% (w/w) of the sample with respect to the potassium bromide (KBr; S.D. Fine Chem Ltd., Mumbai, India) disk. The mixture of the drug and dry KBr was ground into an agate mortar and was compressed into a KBr pellet under a hydraulic press at 10,000 psi. Each KBr disk was scanned 16 times at 4 mm/s at a resolution of 2 cm^−1^ over a wavenumber range of 400–4000 cm^−1^. The characteristic peaks were recorded.

#### Powder X-Ray Diffraction Study

Powder X-ray diffraction (PXRD) patterns of the pure drug, physical mixture of the drug and Eudragit S 100, blank microparticles, and drug-loaded microparticles were investigated using a powder X-ray diffractometer (Philips X’Pert, The Netherlands). An X-ray beam (Philips Cu target X-ray tube) was allowed to fall over the sample. As the slide moved at an angle of theta degree, a proportional detector detected diffracted X-rays at an angle of 2θ° and subsequently, XRD patterns were recorded. XRD patterns were recorded in the 2θ° range of 5 to 60.

### Batch Reproducibility, Packaging, and Stability Study

Three batches of the optimized formulation were prepared and evaluated under the identical conditions. Moreover, the optimized batch was subjected to short-term stability testing according to the ICH guidelines [20]. Microparticles equivalent to 250 mg of LSP were filled in amber-colored glass vials which were further sealed. The sealed vials were exposed to accelerated (40 ± 2°C/75 ± 5% relative humidity) and long-term (25 ± 2°C/60 ± 5% relative humidity) stability for three months. The samples were withdrawn and evaluated for different parameters like visual appearance, powder characteristics, entrapment efficiency, and *in vitro* drug release. DSC was performed at the above-stated time points to observe any changes during storage.

## Results and Discussion

### Formulation Development of Enteric Microparticles of LSP

Enteric polymers exhibit very low solubility in acid and water due to their acidic nature. One of the approaches is to utilize alkaline solutions to render them soluble and acquiescent to spray drying. Thus, we have employed NaOH to solubilize the enteric polymer for the preparation of microparticles [13]. Some preliminary trials were undertaken for the selection of the polymer, appropriate polymer proportions, and basic spray drying conditions for the preparation of microparticles. Preliminary trials for the selection of an appropriate drug:polymer ratio involved spray drying all of the three polymers (Eudragit L 100-55, Eudragit L 100, and Eudragit S 100) starting from a low drug: polymer ratio. Based upon the results of entrapment efficiency and gastric resistance, the ratio was gradually ramped up to observe its effects on the desired quality traits (data not shown). Only the formulations and studies pertinent to the present investigation are presented here. The results are displayed in Table 1. From Table 1 it is clearly stipulated that Eudragit S 100 gave the highest gastric resistance amongst the various Eudragit grades. The reason Eudragit S 100 provided greater gastric resistance is due to a higher pH threshold of the polymer and its polymeric backbone as compared to Eudragit L 100 and Euragit L 100-55. Moreover, the findings also disclose that at a 1:5 drug:polymer ratio, no major difference in entrapment efficiency was observed. Thus, it revealed that entrapment efficiency was independent of Eudragit grades and thus, unaffected by the type of Eudragit.

**Tab. 1:**
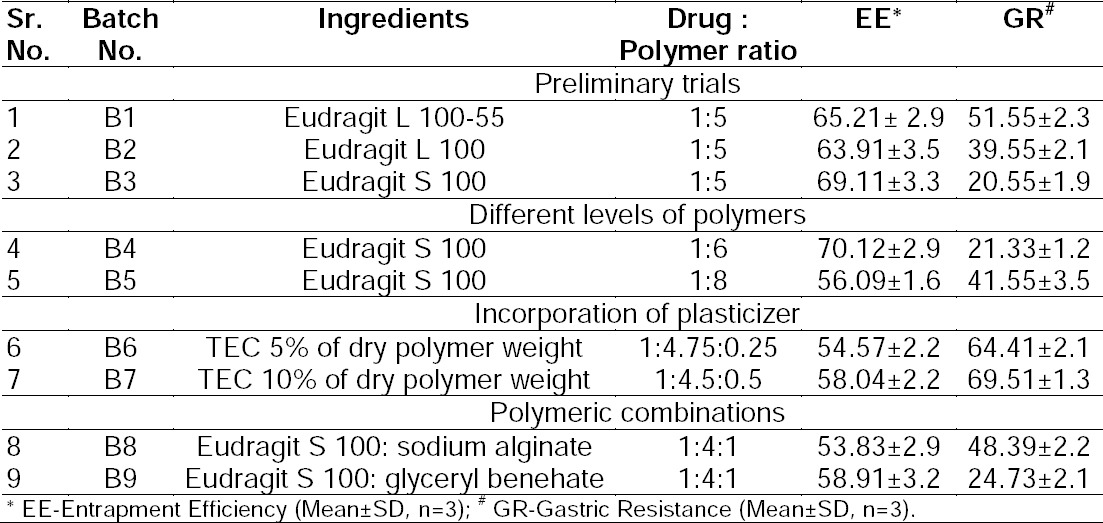
Preliminary trials undertaken for formulation development of lansoprazole enteric microparticles.

In the next step of formulation development, higher levels of Eudragit S 100 were explored viz. 1:6 and 1:8 to investigate its effect on entrapment efficiency and gastric resistance. The results for this step are depicted in Table 1. The results revealed no statistical significance difference (p>0.5) between the drug: polymer ratio of 1:5 and 1: 6, but a significance difference between a 1:5 and 1:8 ratio (p<0.5).

In the next step, plasticizer TEC was incorporated at the 5% and 10% level of the dry polymer. Results are depicted in Table 1. Results revealed that it not only lowered the entrapment efficiency, but also lowered gastric resistance when compared to the polymer alone. The effect of incorporation of TEC was more prominent on gastric resistance rather than on entrapment efficiency. The reason may be that incorporation of the plasticizer lowered the glass transition temperature of the polymer and simultaneously increased the permeability of the microparticle membrane resulting in more ingress of the acidic media inside the microparticles and consequently, augmented drug degradation in acidic media.

Several combinations of Eudragit S 100 were examined with either gastrointestinal tract-insoluble polymers or polymers that swell insoluble in gastric acid pH, but soluble in phosphate buffer pH 7.4. Results disclose that entrapment efficiency was lowered for both sodium alginate and glyceryl behenate, but gastric resistance was diminished more prominently for sodium alginate than for glyceryl behenate (Tab. 1). Ultimately, neither of the polymeric combinations were successful in enhancing gastric resistance more than the polymer alone. Upon looking deeper, this may be due to improper polymer-polymer miscibility resulting in no further improvement in the strength of the microparticle membrane. Another anticipation was made for glyceryl benehate that it would prolong the drug release in phosphate buffer of pH 7.4, but on the contrary, more than 85% drug release was obtained in phosphate buffer of pH 7.4. The reason was that the major portion was Eudragit S 100 which completely dissolved in the buffer due to its pH threshold, which was eventually responsible for complete drug release and subduing the effect of glyceryl benehate.

### Process Optimization for the Spray Drying Process

Spray drying has variegated parameters which need to be optimized to ascertain the desired quality traits. The consequences of the diverse process parameters on the product attributes together with the range of their variation are highlighted in Table 2. Each parameter was varied at a time while keeping others constant, considering their relative impact on product attributes. The value selected for the parameter investigated was carried forward in subsequent trials.

**Tab. 2:**
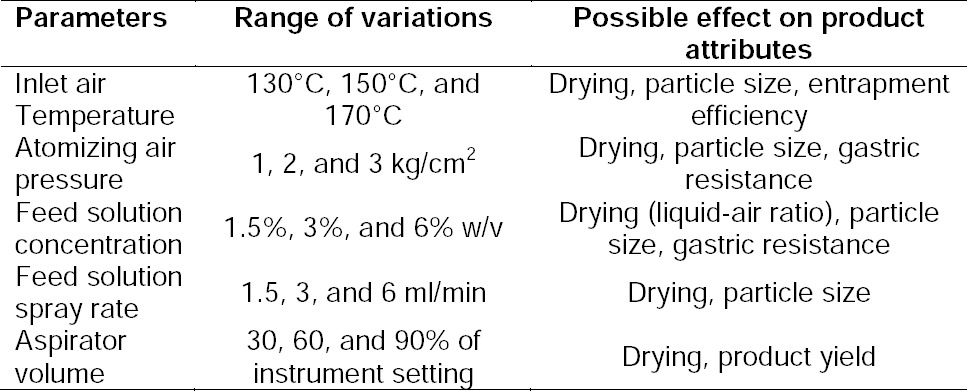
Different levels of process parameters that were varied during spray drying.

#### Inlet Air Temperature

Inlet temperature was varied at three levels: 130°C, 150°C, and 170°C under vacuum. The feed solution concentration was 6% w/v, feed spray rate 3 ml/min, air pressure 2 kg/cm^2^, and aspirator set volume 30%. The inlet air temperature of 130°C resulted in improper drying, leading to maximum deposition of solids on the drying chamber wall instead of accumulating more into the collection vessel. The resultant loss on drying (LOD) (6.98%) was also higher suggesting insufficient drying. When inlet temperature was raised to 170°C, it resulted in the deposition of solids around the mouth of the nozzle, sometimes creating blockage of the nozzle, ultimately creating problems in spray drying. The resultant LOD (1.15%) was relatively satisfactory, but entrapment efficiency was found to be low suggesting possible degradation of the drug due to such a high temperature (Table 3). The inlet temperature of 150°C provided satisfactory drying together with maximum collection in the collector. The LOD (2.12%) was found to be satisfactory and entrapment efficiency was also found to be highest. Gastric resistance was not as much affected as that of entrapment efficiency upon change of the inlet temperature (Table 3). Hence, 150°C inlet air temperature was found to be optimized and was fixed for further experimental trials.

**Tab. 3:**
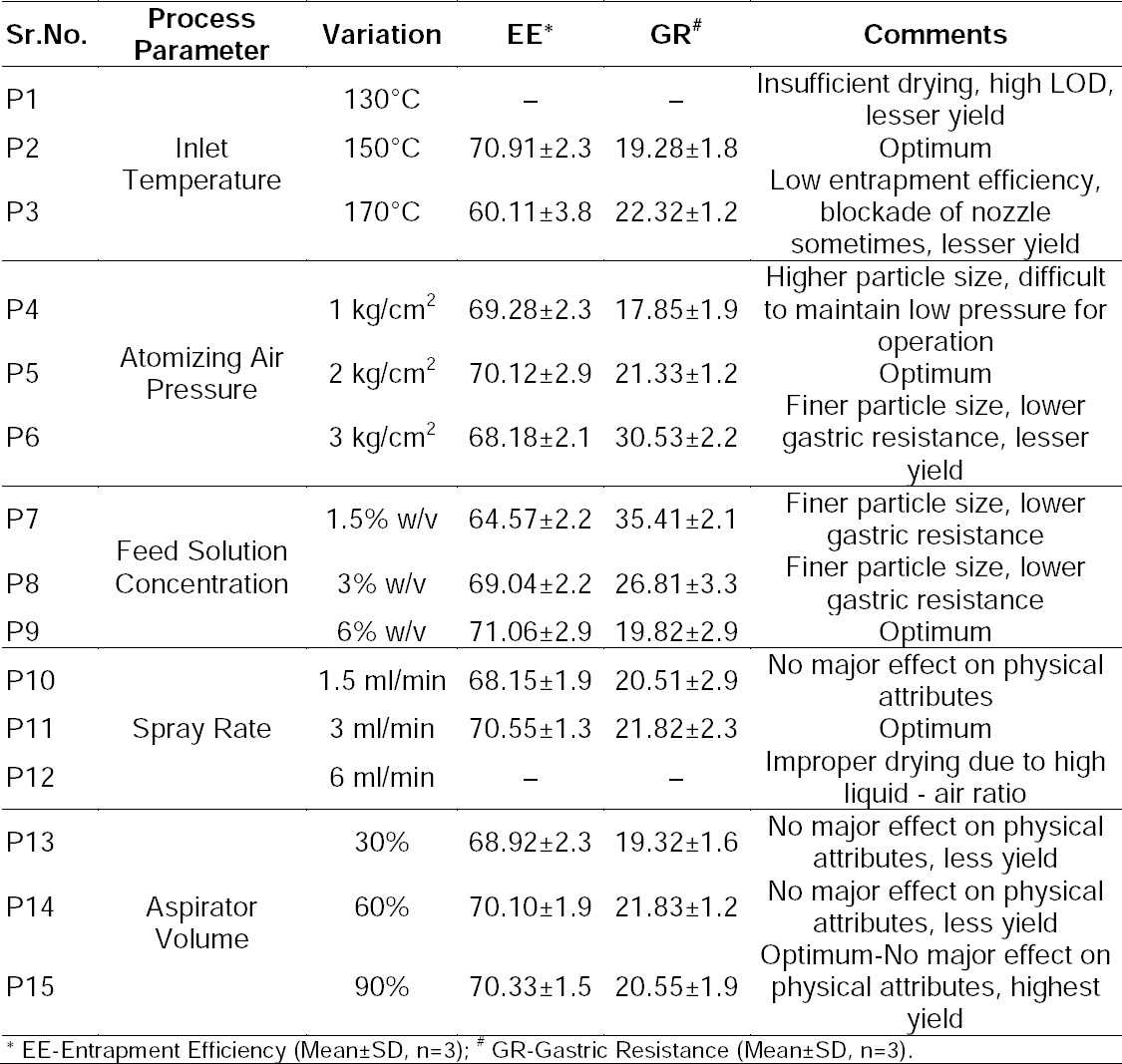
Results of process parameter optimization during spray drying.

#### Atomizing Air Pressure

Air pressure was varied at 1, 2, and 3 kg/cm^2^ with inlet air temperature of 150°C, and all other parameters were as previously mentioned. High pressure resulted in fine particles of size 0.5–1.8 µm. It did not affect entrapment efficiency, but rather gastric resistance was found to be lower (Table 3). Also, the proportion of product deposited into the scrubber was higher than into the collection vessel. The reason for lower gastric resistance may be the higher surface area provided by the finer size which allowed more acidic media to diffuse in the microparticles resulting in lower gastric resistance. Thus, the results were contrary to the ideal characteristics of microparticles. The lower air pressure of 1 kg/cm^2^ resulted in relatively larger size particles of 9–12 µm with higher gastric resistance (Table 3), but maintenance of such a low pressure was difficult due to technical limitations. The air pressure of 2 kg/cm^2^ provided particle sizes of around 4–7 µm with higher gastric resistance with no problems in machine operation (Table 3). Regardless of the air pressure, cohesive powder was obtained with poor flow properties at all the particle size ranges. The atomizing air pressure was kept at 2 kg/cm^2^ for further experimentation.

#### Feed Solution Concentration

The feed solution concentration was varied at three levels: 1.5%, 3%, and 6% w/v. Lower concentrations resulted in finer particles (0.6–1.2 µm) which may be due to slower evaporation of the solvent leading to slower formation of the solid wall of microparticles. Additionally, as discussed earlier, gastric resistance was found to be less and there was more deposition of solids into the scrubber due to fine particle size. Upon increasing the feed concentration to 3% w/v, there was improved gastric resistance. Upon further increasing the feed solution concentration to 6%, the particle size (4–7 µm) simultaneously increased and provided higher gastric resistance. This may be due to faster drying of particles with higher solid content. The higher solid concentration leads to the formation of a crust of solid faster upon solvent evaporation as compared to lower solid concentration. Entrapment efficiency was not affected by feed solution concentration (Table 3). Thus, the feed solution concentration was kept at a maximum level of 6% w/v for further experimentation. Moreover, a higher solid content permits saving valuable time, energy, and solvent.

#### Feed Solution Spray Rate

The spray rate was varied at 1.5, 3.0, and 6 ml/min, with the other parameters as optimized previously and aspirator setting at 30%. The higher spray rate of 6 ml/min resulted in insufficient solvent evaporation due to high liquid-air ratio. This led to the deposition of spray mist on the wall of the drying chamber and minimal deposition of the solid into the collection vessel. On the contrary, the lower spray rate of 3 ml/min and 1.5 ml/min resulted in maximum deposition of solids into the collector vessel with minimal differences in its physical properties and gastric resistance. Thus, the spray rate of 3 ml/min was fixed due to lower time consumption than 1.5 ml/min with the desired product attributes.

#### Aspirator Volume

The aspirator settings were kept as 30%, 60%, and 90% of the instrument settings considering the lower, middle, and maximum instrument settings range for continuous operation. The results revealed that the higher the aspirator settings, the higher the product recovery. The yield proportionately increased with the aspirator volume. It did not impact other product physical attributes and gastric resistance. Hence, the aspirator setting was fixed at 90% of the instrument setting.

### In Vitro Drug Release

**Fig. 1:**
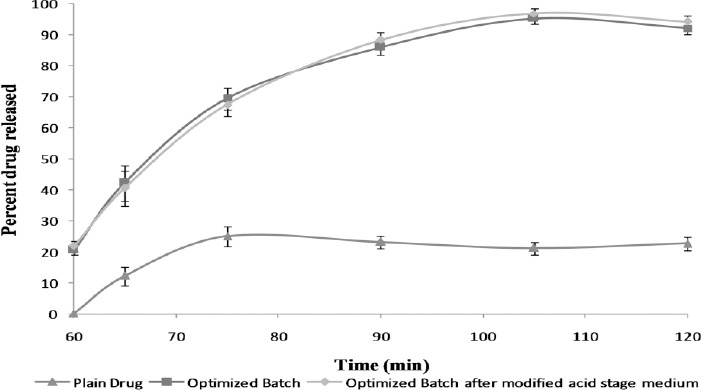
Dissolution profile in 900 ml, USP Apparatus-II, 75 rpm, phosphate buffer pH 7.4 for the plain drug, optimized batch and optimized batch after modified acid stage medium.

*In vitro* drug release of the drug remaining stable after exposure to acidic media was carried out in 900 ml phosphate buffer of pH 7.4. All the batches showed complete drug release of more than 85% in the medium selected. The *in vitro* drug release of the optimized batch B3 with process parameters as optimized above is highlighted in Fig. 1. The results revealed more than 85% drug release from the formulation with a low standard deviation amongst different units while incomplete release was observed from the plain drug. Thus, the formulation diminished not only the variability reasons due to its solubility, but also increased the solubility of the drug due to its amorphous nature.

### Gastric Resistance and In Vitro Drug Release in Modified Acid Stage Medium pH 4.5

As discussed earlier, *in vivo* pH of patients on multiple dose regimens of PPI is >4, thus microparticles were tested in the modified acid stage medium of pH 4.5 which better simulates the gastric environment. Gastric resistance in pH 4.5 acetate buffer was found to be 21.82 ± 1.6 which was not significantly different from gastric resistance in 0.1 N hydrochloric acid (HCl). The *in vitro* drug release in phosphate buffer of pH 7.4 is depicted in Fig. 1. From Fig. 1, it can be observed that more than 85% of the drug was released in pH 7.4 phosphate buffer media.

### Characterization of Microparticles

#### DSC Study

**Fig. 2:**
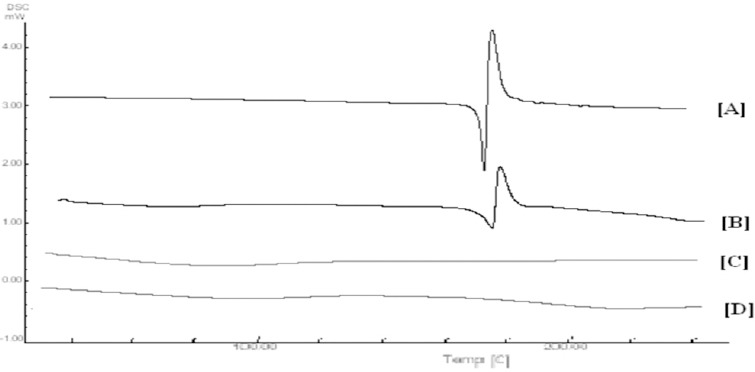
DSC spectra for [A] LSP, [B] Physical mixture, [C] Blank microparticles, and [D] Drug-loaded microparticles.

DSC spectra of LSP, the physical mixture of the drug and polymer, blank microparticles, and drug-loaded microparticles are depicted in Fig 2. DSC analysis of LSP exhibited two different events: an endothermic peak at 177.41°C corresponding to the melting point of the drug which was immediately followed by an exothermic peak corresponding to the degradation of the drug. Physical mixtures of the drug and polymer sample showed an endothermic peak at 70.20°C correlating to the polymer and the other one at 178.32°C correlating to the LSP. Moreover, the exothermic peak correlating to the degradation of LSP was also observed. In the case of microparticles, complete absence of the endothermic as well as the exothermic peak of LSP was depicted signifying that the drug is molecularly dispersed within the polymer, stabilizing the effect on the drug and restraining its degradation. The thermal behavior of drug-loaded microparticles was similar to the blank micorparticles, thus supporting the above hypothesis.

#### FTIR Study

**Fig. 3:**
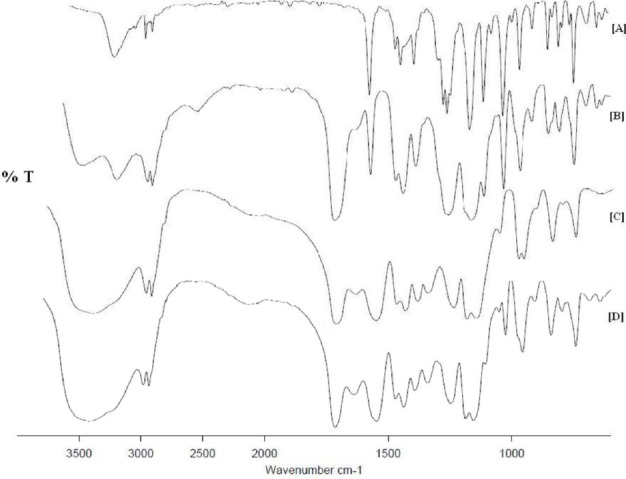
FTIR spectra for [A] LSP, [B] Physical mixture, [C] Blank microparticles, and [D] Drug-loaded microparticles.

During preparation of the microparticles, there are chances for interaction between the drug and the polymer. As we know, enteric Eudragit polymers contain acidic functional groups and if they interact with the drug, it can lead to the degradation of it. To investigate any lack of interaction between the drug and polymer, FTIR spectroscopy was used. The FTIR spectra of the drug, physical mixture, blank microparticles, and drug-loaded microparticles are displayed in Fig 3. The FTIR spectra of LSP exhibited characteristic peaks at 3238, 2983, 1581, 1476, 1283, and 1117, signifying stretching vibrations of –NH, –CH_2_-, aromatic ring, –NH bending, C-N of the pyridyl ring, and the ether bond, respectively [10]. The polymer sample exhibited the C=O vibrations of the carboxylic acid groups at 1705 cm^−1^ and at 1730 cm^−1^ for the esterified carboxylic acid group. When the polymer was neutralized with NaOH, its ionization occurred and the characteristic peak of the carboxylic acid group at 1705 cm^−1^ was replaced by a band at 1560 cm^−1^ corresponding to the anti-symmetrical vibration of the –COO^−^ [13]. These changes were seen in both the blank and drug-loaded microparticles. The esterified carboxylic acid peak remained at the same position at 1730 cm^−1^, signifying that it did not involve any type of interaction. Now, the FTIR spectra of the drug-loaded microparticles was compared with the FTIR spectra of the physical mixture and plain drug. The spectra did not show any shift in its characteristic peaks of LSP in microparticles, suggesting no new chemical bond formation. Thus, this observation ruled out the possibility of an interaction between the drug and polymer indicating that the LSP was physically dispersed in the Eudragit S 100.

#### PXRD Study

**Fig. 4:**
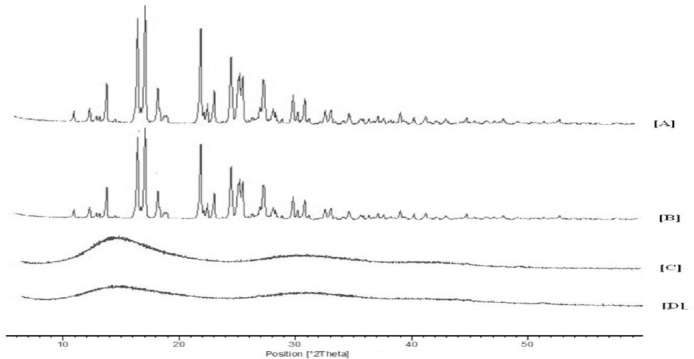
PXRD spectra for [A] LSP, [B] Physical mixture, [C] Blank microparticles, and [D] Drug-loaded microparticles.

PXRD diffractograms of pure LSP, the physical mixture, blank microparticles, and drug-loaded microparticles are depicted in Fig. 4. The variegated distinct peaks appearing in the diffractogram discloses that LSP is in crystalline form with characteristic peaks at an angle of 2θ at 11.40, 12.76, 14.24, 16.90, 17.50, 18.62, 22.29, 24.90, and 27.73. Blank microparticles were characterized by a halo pattern or complete absence of any peaks in the diffractogram. The XRD peaks of LSP were found in the same position in the physical mixture while complete absence was seen in the drug-loaded microparticles, indicating amorphization of the drug. The results were in line with the DSC data, confirming amorphization of the drug in the microparticles.

#### SEM Study and Powder Characteristics

**Fig. 5:**
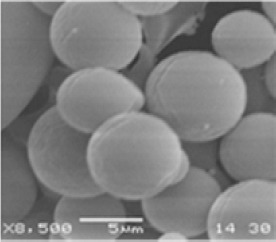
Topographical images of microparticles carried out by SEM.

The results of the SEM analysis are demonstrated in Fig. 5. The results disclose that the microparticles represented spherical and smooth surfaces. The powder characteristics of the optimized batch B3 is depicted in Table 4. From Table 4, results revealed that microparticles had poor flow according to Carrs’s and Hausner’s ratio comparison. Moreover, bulk density was found to be higher as small particles are usually more dense and exhibit higher bulk density. Additionally, irrespective of the process conditions, all the batches exhibited poor flow characteristics of the powder.

**Tab. 4:**
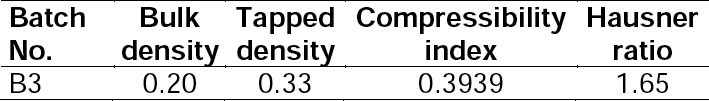
Powder characteristics of microparticles for the optimized batch.

### Product Suitability

In the research envisaged, enteric microparticles were prepared in a single step by spray drying technology. Employing this technique facilitated formulation development in a ready-to-formulate form. The microparticles resulted in significant enhancement of gastric resistance (Table 1) together with improved solubility in the dissolution medium (Fig. 1). The microparticles can be directly blended with fillers, glidants, and lubricants and filled directly into hard gelatin capsules. Thus, it reduces overall processing time and augments formulation productivity.

### Batch Reproducibility and Stability Studies

The three batches of optimized formulation B3 were prepared and evaluated for physicochemical properties under identical experimental conditions. The entrapment efficiency, gastric resistance, and *in vitro* drug release did not show significant differences between the three sets of batches revealing the reliability and reproducibility of the manufacturing process.

At all of the sampling times in the stability studies including the completion of stability studies, the microparticles demonstrated shape and powder characteristics similar to those of the initial samples. Regarding the DSC study, the peak of LSP was not observed for both the initial and 3-month stability samples. The spectra did not show any differences upon comparison and moreover, no additional peaks were observed. The results of the stability data are displayed in Table 5. The results demonstrated no significant changes in entrapment efficiency and gastric resistance for the initial, long–term, and accelerated stability samples.

**Tab. 5:**
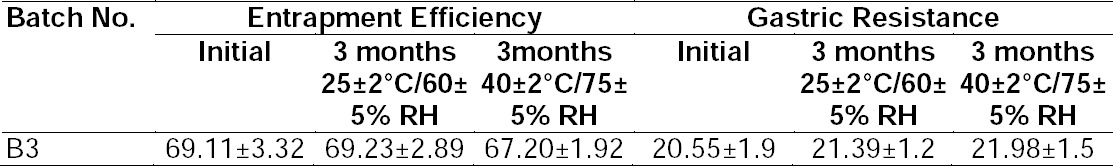
Stability study data for the optimized batch.

## Conclusion

With mounting awareness of spray drying techniques for the preparation of drug microparticles commercially, the usefulness of it has now prudently entered into the realms of research and industry for understanding the process or formulation variable rationally. This manuscript describes the development and optimization of enteric microparticles of LSP using spray drying. In an endeavor to accomplish the objectives, preliminary trials were undertaken to screen the types of Eudragit and its levels. Eudragit S 100 was found to be the optimized polymer at the drug:polymer ratio of 1:5 with gastric resistance around 20% and more than 85% drug release in phosphate buffer of pH 7.4. The gastric resistance was not affected by modified acid stage medium of pH 4.5 acetate buffer. Finally, the process parameters were optimized to get reproducible results. The optimized process was comprised of inlet temperature of 150°C, atomizing air pressure of 2 kg/cm^2^, feed solution concentration of 6% w/w, feed solution spray rate of 5 ml/min, and aspirator volume of 90%. Microparticles by SEM revealed smooth surfaces and spherical morphology. DSC, PXRD, and FTIR confirmed that LSP was physically dispersed in the polymer. The product was found to be stable over 3 months accelerated and long-term conditions as per ICH Q1A(R2) guidelines.

Hence, the developed microparticles of LSP can provide a better approach for enteric delivery in terms of the formulation aspect that can be directly filled into capsules with nominal usage of excipients. Moreover, the manufacturing method employed can be easily adopted in industries.
